# Effects of Menthol Mouth Rinsing on Performance and Surface EMG Activity During Heat-Stressed Cycling

**DOI:** 10.3390/nu18071134

**Published:** 2026-04-01

**Authors:** Kierstyn V. Hawke, Ryan C. A. Foley, Nicholas J. La Delfa, Heather M. Logan-Sprenger

**Affiliations:** Faculty of Health Sciences, Ontario Tech University, Oshawa, ON L1G 0C5, Canada; ryan.foley@ontariotechu.ca (R.C.A.F.); nicholas.ladelfa@ontariotechu.ca (N.J.L.D.)

**Keywords:** menthol, thermal stress, performance enhancement, neuromuscular fatigue, adolescents

## Abstract

**Objective:** This study investigated the effects of menthol (MEN) mouth rinsing (MR) on cycling performance, neuromuscular activation, and perceptual responses during high-intensity exercise in the heat. **Methods:** A total of 10 trained adolescent male cyclists (16.7 ± 1.3 yrs; VO_2peak_: 62.3 ± 7.6 mL·kg^−1^·min^−1^) completed a familiarization and two randomized, single-blind trials using a modified variable cycling test (M-VCT) in the heat (31.45 ± 0.59 °C; 23.40 ± 2.55% RH). The participants rinsed with 0.01% L-menthol or a placebo every 6 min during exercise. Power output (PO), cadence (RPM), rating of fatigue (ROF), affective feeling (FS), and surface electromyography (sEMG) were recorded. **Results:** Menthol MR significantly increased mean PO by 1.67 ± 1.59% (MEN: 177.1 ± 33.0 W; PLA: 174.1 ± 32.1 W; *p* = 0.002; *d* = 1.42) and enhanced cadence (MEN: 87.4 ± 5.1 RPM; PLA: 84.5 ± 5.2 RPM; *p* = 0.027; *d* = 0.84), particularly during high-intensity intervals. No significant differences were observed in ROF or FS between conditions (*p* > 0.05). Five muscles were monitored for activation (RF, VM, VL, TA, Gast). A significant main effect of time demonstrated decreased activation in VM, TA, RF, and Gast. VL showed a trend toward a main effect of condition (*p* = 0.057), with lower activation in MEN. Both VL and RF exhibited significant condition × lap interactions (*p* = 0.007 and *p* = 0.017), with progressively lower activation in MEN as fatigue progressed. **Conclusions:** Menthol MR significantly improved cycling performance in the heat without altering perceptual or physiological strain. Some muscles demonstrated reduced activation with menthol MR, and further study is needed to confirm the magnitude of ergogenic effects and elucidate the physiological mechanism.

## 1. Introduction

Cycling is a metabolically demanding sport requiring sustained power output and neuromuscular coordination, with performance ultimately constrained by fatigue—a multifactorial phenomenon involving both peripheral and central mechanisms [1]. Exercise-induced fatigue (EF) is defined as a reversible decline in the ability to generate force or power [2], arising either from peripheral impairments, such as disrupted excitation-contraction coupling, or from central limitations in motor drive [3]. In cycling, peripheral fatigue tends to dominate short, intense efforts (e.g., 4 km time trials), while central fatigue becomes more influential during prolonged submaximal tasks [4]. Although fatigue serves a protective role by maintaining physiological stability, competitive athletes continually pursue strategies to attenuate its impact and sustain performance [5]. Furthermore, performance capacity in young, trained athletes is of particular interest, as it has been suggested that fatigue may influence cycling dynamics differently between adults and adolescents depending on training and experience [6]. A cohort of trained adolescent males is of particular interest, as it reflects athletes transitioning toward elite competition yet remains underrepresented in the literature.

Surface electromyography (sEMG) has emerged as a widely accepted, non-invasive method for quantifying muscle recruitment and fatigue by assessing changes in amplitude and frequency characteristics of muscle action potentials [7,8,9]. By measuring muscle fibre conduction velocity (CV) and action potential characteristics, sEMG provides valuable insight into both the temporal and spatial aspects of muscle fatigue [3]. In cycling-specific contexts, sEMG enables the evaluation of coordinated lower limb muscle activation, such as the vastus lateralis, rectus femoris, gastrocnemius, and tibialis anterior, responsible for power transfer throughout the pedal stroke [10,11,12]. Environmental stressors, particularly heat, further compound the development of fatigue. Exercising in hot conditions accelerates the onset of fatigue through mechanisms such as hyperthermia, dehydration, and cardiovascular drift, which collectively impair both central and peripheral function [13]. As core temperature increases, thermal strain may limit neural drive to the working muscles, compromise thermoregulatory efficiency, and elevate perceived exertion, ultimately impairing performance [5,14].

Menthol (MEN), a compound known for its potent cooling sensation, has gained interest as a non-thermal, sensory-based ergogenic aid [15,16]. Under heat stress, menthol activates transient receptor potential melastatin 8 (TRPM8) ion channels in oral and dermal tissues, eliciting a perception of coolness without lowering core body temperature [15]. This sensory effect can reduce thermal discomfort and perceived exertion, potentially sustaining effort under heat stress [17]. Prior research has demonstrated that menthol application (topical) or mouth rinsing (MR) can improve performance in hot environments by modulating thermal perception [16], although the underlying neuromuscular mechanisms still remain unclear. In our previous work, we demonstrated that menthol mouth rinsing significantly enhanced cycling performance during a simulated race task in the heat, yet the physiological basis for this ergogenic effect—particularly regarding muscle activation—was not examined [17]. Given the interplay between perception, neuromuscular fatigue, and environmental stress, further exploration into the influence of menthol on central and peripheral fatigue pathways is warranted.

The purpose of this study was to evaluate the effects of repeated menthol mouth rinsing on cycling performance and neuromuscular activation in trained adolescent male cyclists exercising under heat stress. By incorporating sEMG measurements from key locomotor muscles in the lower limbs, we aimed to elucidate the extent to which menthol MR modifies muscle recruitment patterns and fatigue development. We hypothesized that menthol MR would improve performance, which is reflected by increased work output with similar or reduced muscle activation. These findings may inform the application of menthol-based strategies for enhancing endurance performance in hot competitive environments.

## 2. Materials and Methods

### 2.1. Participants

The participants were recruited using the same inclusion criteria as our previous study [17]. Ten (*n* = 10) trained adolescent males aged between 15 and 19 yrs (age 16.7 ± 1.3 yrs, body mass 65.6 ± 12.3 kg, height 176.8 ± 9.3 cm, average weekly training 11.9 ± 3.4 h, VO_2peak_ 62.3 ± 7.6 mL·kg^−1^·min^−1^) volunteered. A male-only sample was selected to reduce biological variability and provide a more controlled initial examination of MEN MR and EMG activity in this population. This study was conducted according to the guidelines of the Declaration of Helsinki and approved by the Research Ethics Board of Ontario Tech University (REB#16331, 22 December 2021).

### 2.2. Study Design

A single-blind, randomized, crossover design replicated previously described methodology [17]. The participants completed a maximal incremental test, familiarization, and two experimental M-VCT trials using menthol (MEN) or placebo (PLA) MR, conducted at the same time of day and on the same ergometer (LODE Excalibur Sport, Quinton Instrument, Groningen, The Netherlands). The sessions were separated by 7 days to ensure adequate recovery between efforts. Environmental conditions were tightly controlled and replicated across trials. Hydration status was standardized before each session, and sweat loss was monitored to confirm comparable thermoregulatory strain between conditions.

### 2.3. Testing Protocols

Visit one included anthropometric and preliminary measurements (body mass, height, and USG) and a maximal incremental test to determine VO_2peak_ and peak power output (PPO). On the second visit, participants completed an M-VCT heat familiarization session (31.2 ± 0.8 °C, 23.0 ± 2.0% RH) to minimize learning effects [18]. Previous work has demonstrated that the M-VCT protocol has a reliability of 0.46% between test–retest sessions in trained cyclists [17]. In the following sessions, two M-VCT experimental sessions were conducted in the heat (31.4 ± 0.9 °C, 23.4 ± 3.7% RH) (see Hawke et al. 2025 [17] for the complete M-VCT protocol). Standardized verbal encouragement and instruction were used in all experimental trials. The participants received verbal and visual instruction on how to subjectively assess rating of fatigue (ROF) on a 0–10-point scale [19] and affective feeling (FS) from an 11-point scale (−5 to +5) [20].

Power output (W) and cadence (RPM) were recorded continuously, whereas ROF and FS were collected every 6 min, following mouth rinsing. sEMG (Delsys Trigno, Natick, MA, USA) was collected continuously from five muscles on the right limb to assess muscle activation [9]. At six time points during the M-VCT (beginning of each lap), participants swilled a volume- (25 mL), colour- (blue non-caloric edible food colouring, Club House, McCormick & Company, Commerce, CA, USA), and temperature-matched (warmed to >22 °C) mouth rinse of either 0.01% L-menthol solution ([0.64] mM) (Sigma Aldrich, Merck KGaA, Darmstadt, Germany), formulated from 0.1 g of MEN crystals dissolved in 1 L of deionized water heated to 40 °C [21,22,23], or a PLA solution made using deionized water and a non-caloric berry-flavoured sucralose sweetener (Crystal Light, Don Mills, ON, Canada) [24].

### 2.4. Surface Electromyography (sEMG) Measurements

**Figure 1:**
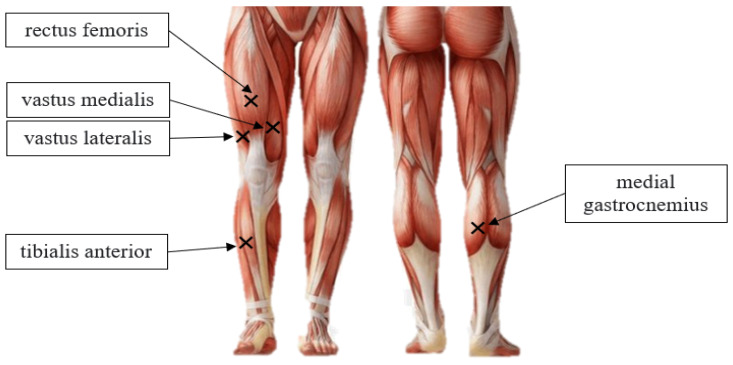
Identification of sEMG sensor sites on the right limb. Anterior muscles include rectus femoris (RF), vastus medialis (VM), vastus lateralis (VL), and tibialis anterior (TA). Posterior muscle is the medial gastrocnemius (Gast). Image adopted and modified [30].

sEMG was used to assess neuromuscular function throughout the M-VCT. Five leg muscles were monitored using wireless bipolar electrodes from a Delsys Trigno EMG system (Natick, MA, USA) [9]. Sensors were placed unilaterally on the right vastus lateralis (VL), vastus medialis (VM), rectus femoris (RF), tibialis anterior (TA), and medial gastrocnemius (Gast) (Figure 1). Prior to mounting the bike, while in a seated position, each participant had the placement sites for the five electrodes prepared in accordance with the SENIAM guidelines [25]. First, muscle location(s) was identified via measurement and landmarked on the skin. Next, the area was dry-shaved with a disposable razor and cleaned with an alcohol swab to remove contaminants on the skin that could affect impedance. Each sensor was affixed using proprietary Delsys adhesive pads and additionally secured using Hypafix tape (Leukoplast Inc., Stockholm, Sweden).

The signals were sampled at 2000 Hz through the Delsys EMGworks software, version 4.8 (Natick, MA, USA) before export for analysis in a custom LabVIEW programme, version 22.3 (National Instruments, Austin, TX, USA). The data were band-pass filtered with a 4th-order Butterworth filter from 20 to 500 Hz. The EMG amplitude (aEMG) signals were smoothed using a 0.15 s RMS window applied over the duration of the time trial or sprint. aEMG was normalized to each participant’s individual peak activation level from the entire session, including laps and sprints [26]. Thus, all sEMG measures are reported as percentage of peak (% peak EMG). Muscle activation during cycling is rhythmic, increasing and decreasing during the push and pull phases, depending on the muscle examined and the timing of the pedal stroke [27,28]. Thus, to avoid phase sampling effects, sEMG signals were collected and averaged over a 5 s window at the midpoint of each fixed workload (3.5 W/kg) per lap. This averaging has previously been noted as a desirable way to investigate slow time-series and fatigue-related changes rather than intra-limb activation times [29]. This processing resulted in 30 discrete EMG measures (six per lap, five laps) per participant, collected at equivalent workloads to examine sEMG amplitude for fatigue-related changes and the effects of menthol MR supplementation.

## 3. Statistical Analysis

The cycling performance, physiological, and perceptual data were complete across all participants and conditions, permitting analysis via standard parametric tests. The EMG data contained missing observations due to technical issues, necessitating a separate linear mixed-effects model approach to retain all available data from the small, specialized sample. The data are reported as means ± SD in the text. For greater readability, figures display means or estimated marginal means ± SEM, as noted in figure captions.

### 3.1. Cycling, Physiological, and Subjective Measures

Statistical analysis was done using SPSS IBM, version 28 (SPSS Inc., Chicago, IL, USA). All variables were screened for normality using the Shapiro–Wilk test, z-score for skewness, and kurtosis of ±3. Paired-samples *t*-tests were used to determine single-parameter differences between variables. Two-way repeated-measures ANOVA was used to assess the effects of time (lap) and condition and the time (lap) × condition interaction. In the event of a significant effect, further pairwise comparisons were made using a Bonferroni post hoc correction factor to determine significant differences. Time (lap) versus condition ordinal data were evaluated using the Friedman test, and singular differences were detected using the Wilcoxon Signed Ranks Test set at *p* = 0.008. For all statistical tests, significance was assumed at an alpha level of <0.05, and 95% confidence intervals of the mean difference were reported. Effect sizes for paired *t*-tests are reported as standardized mean differences (Cohen’s *d*) and interpreted based on Cohen’s criteria: 0.2 = small, 0.5 = moderate, 0.8 = large effect [31]. Effect sizes for ANOVA models are reported as partial eta squared (η*p*^2^), and reference values were interpreted as 0.01 = small, 0.06 = moderate, and 0.14 = large effect [31].

### 3.2. EMG Measures

Upon study completion, some sEMG data were missing (4.7% of measurements) due to technical sampling issues and electrode battery failure or suspected normalization-induced outliers. Thus, the data were deemed missing not at random and were therefore ineligible for synthetic replacement via multiple imputation methods. Instead, it was determined that the best approach to retain data while still statistically evaluating the effect of menthol MR on sEMG measures was to mark these data as N/A and use mixed-effects models [32]. There was no anticipated muscle-specific interaction with the conditions, and each muscle was modelled and tested independently. Therefore, selected fixed effects were condition (two levels), lap (five levels), and workload (five levels) with a random effect of participant (*n* = 10). All statistical analyses for the EMG data were performed using R (v4.5.1) in the RStudio (v2023.06) statistical software. Each model was also fitted with a maximal random effects structure, both random slopes and random intercepts for all within-subject factors, using the *buildmer* R package [33]. All muscles converged on the same random effects structure. Each model was visually examined to confirm the normal distribution of residuals and that homoscedasticity assumptions were not violated. The data were processed as normalized EMG and were not transformed prior to testing. Each model was examined with independent ANOVA using a Kenward–Roger degrees of freedom method [34], and significance was set at *p* < 0.05. Post hoc comparisons were examined only for statistically significant results using estimated marginal means and a Tukey method multiple comparison correction [35].

## 4. Results

### 4.1. MEN/PLA Experimental Sessions in the Heat

All data were normally distributed, and no order effect was observed between first and second trials (*p* = 0.763). No significant differences existed in environmental conditions between the MEN and PLA trials (MEN, 31.45 ± 0.59 °C, PLA, 31.35 ± 0.64 °C, *p* = 0.547, 95% CI = [−0.26 to 0.46], *d* = 0.20; MEN, relative humidity 23.40 ± 2.55%, PLA, 23.30 ± 2.91%, *p* = 0.847, 95% CI = [−1.04 to 1.24], *d* = 0.06). Additionally, there was no significant difference in mouth rinse temperature between conditions (MEN, 25.38 ± 1.07 °C, PLA, 25.85 ± 1.78 °C, *p* = 0.182, 95% CI = [−1.60 to 0.66], *d* = 0.370).

All athletes arrived euhydrated with USG < 1.020 (MEN, 1.004 ± 0.003; PLA, 1.006 ± 0.005; *p* = 0.051, 95% CI = [−0.006 to 0.0002], *d* = 0.71). Total body sweat loss was not significantly different between conditions (MEN, 0.91 ± 0.44 L; PLA, 0.83 ± 0.40 L, *p* = 0.235, 95% CI = [−0.06 to 0.22], *d* = 0.40.

### 4.2. M-VCT Performance and Menthol Mouth Rinsing

See Table 1 and Table 2 for a summary of trial comparisons for MEN versus PLA.

**Figure 2:**
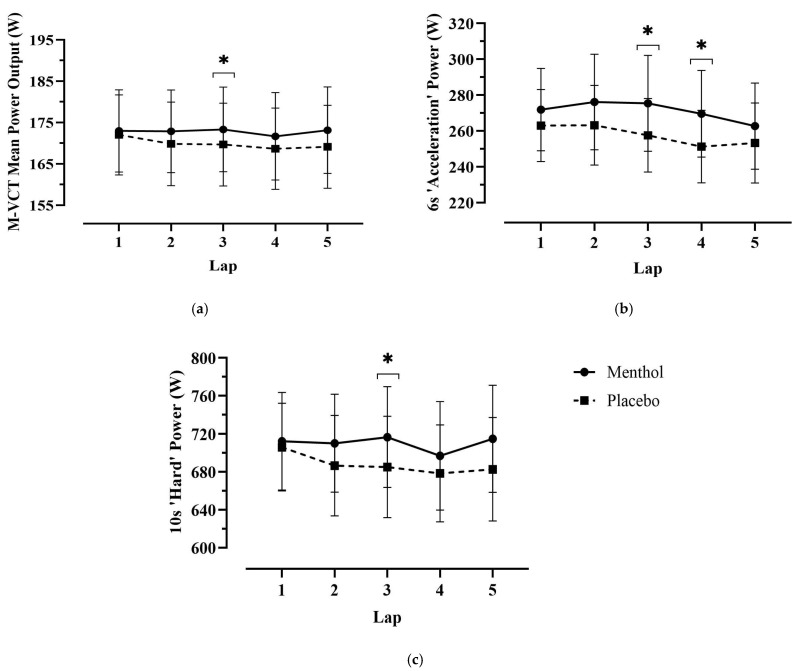
Mean power output (PO) during key performance sections per lap of the M-VCT, comparing MEN versus PLA. Data are presented as means ± SEM (*n* = 10). (**a**) Mean PO was significantly greater in the MEN trial during lap 3 (*p* = 0.008) when compared to PLA. (**b**) PO during 6 s “acceleration” was significantly greater in the MEN condition during laps 3 and 4 (*p* = 0.029, *p* = 0.030) and (**c**) PO during 10 s “hard” was significantly greater in the MEN condition during lap 3 (*p* = 0.019). * Significantly greater in the MEN trial (*p* < 0.05).

Mean power output (POmean) for the entire M-VCT was significantly higher in the MEN condition by 1.67 ± 1.59% (corrected based on coefficient of variation (CV)) when compared to PLA (Table 1). Mean 10 s (hard) power output was also significantly higher for MEN (MEN, 709.4 ± 166.3 W; PLA, 687.3 ± 159.0 W, *p* = 0.004, 95% CI = [8.79 to 35.35], *d* = 1.19), in line with findings by Hawke et al. 2025 [17] (Figure 2).

### 4.3. Peak Power Output (PPO)—1 s

While absolute 1 s peak power output across the entire trial (PPOtrial) was slightly higher for MEN, the difference was not significant. PPO was also not significantly different during “acceleration” (PPO6), “hard” (PPO10), or the final sprint (PPOsprint) between conditions (Table 1).

### 4.4. Cadence (RPM)

**Figure 3:**
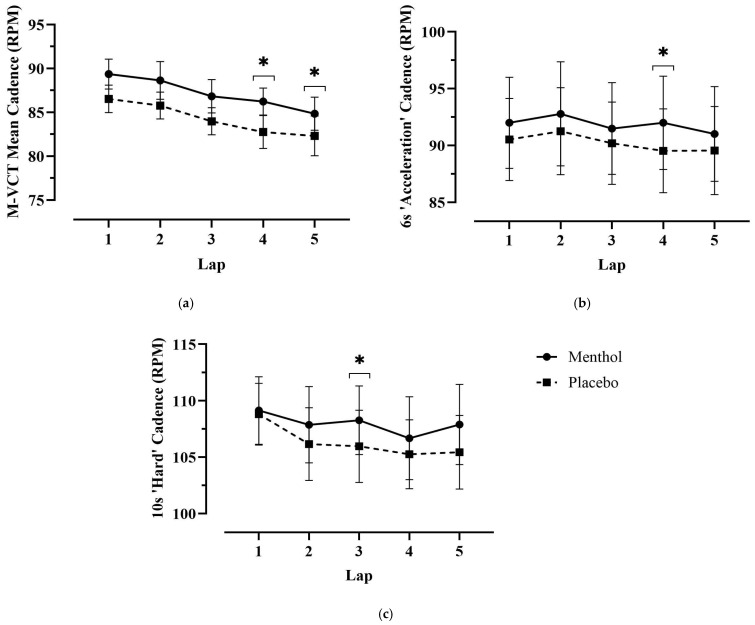
Mean cadence (RPM) during key performance sections per lap of the M-VCT, comparing MEN versus PLA. Data are presented as means ± SEM (*n* = 10). (**a**) Mean RPM was significantly greater in the MEN trial during lap 4 (*p* = 0.005) and lap 5 (*p* = 0.05) when compared to PLA. (**b**) RPM during 6 s “acceleration” was significantly greater in the MEN condition during lap 4 (*p* = 0.050), and (**c**) RPM during 10 s “hard” was significantly greater in the MEN condition during lap 3 (*p* = 0.034). * Significantly greater in the MEN trial (*p* < 0.05).

Although average cadence declined over time in both conditions (*p* > 0.05), the total mean cadence (RPMavg) was significantly greater in the MEN condition compared to PLA (Table 1). Lap-wise comparisons revealed significantly higher cadence during lap 4 (*p* = 0.005) and lap 5 (*p* = 0.050) in the MEN trial. Furthermore, average cadence during both the “acceleration” (RPMavg_6) and “hard” (RPMavg_10) segments of the trial was higher in the MEN condition. Specifically, during the acceleration phase, cadence was 92.4 ± 12.4 RPM in MEN versus 90.6 ± 11.2 RPM in PLA (*p* = 0.078; 95% CI: [−0.25 to 3.92]; ES = 0.63), and during the hard phase, cadence was significantly higher, at 107.4 ± 10.0 RPM in MEN compared to 106.0 ± 9.4 RPM in PLA (*p* = 0.022; 95% CI: [0.26 to 2.66]; ES = 0.87). Post hoc analyses further indicated a significantly higher lap-specific cadence in the MEN trials for 6 s “acceleration” sections (RPMlap_6s) during lap 4 (*p* = 0.050) and for 10 s “hard” sections (RPMlap_10s) during lap 3 (*p* = 0.034) (Figure 3). No significant difference in cadence during the final sprint was observed between conditions. Overall, menthol MR significantly enhanced cadence maintenance, particularly in the later stages of the M-VCT, despite reductions in neuromuscular activation and higher power output demands.

### 4.5. Cardiovascular and Thermoregulatory Responses

**Figure 4:**
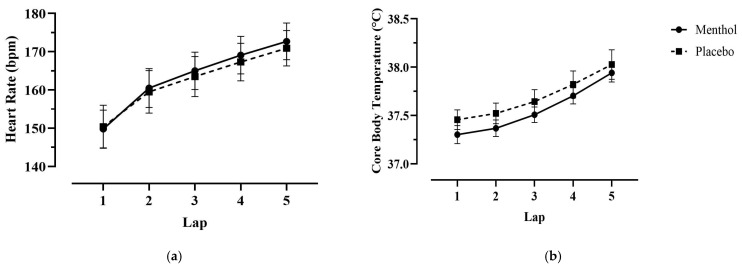
Relationship between heart rate (HR) (**a**), core temperature (Tc) (**b**), and laps completed in the modified variable cycling test (M-VCT). Data are presented as means ± SEM (*n* = 10). HR increased over time in both conditions, but no significant difference was found between trials (*p* = 0.373). TC increased over time in both conditions, but no significant difference was found between trials (*p* = 0.237).

HR and Tc increased significantly across exercise time in both trials (HR: *p* < 0.001, η*p*^2^ *=* 0.894; Tc: *p* < 0.001, η*p*^2^ *=* 0.859). There was no significant difference in mean or max HR or Tc between trials (Table 1, Figure 4).

### 4.6. Perceptual Scales

**Figure 5:**
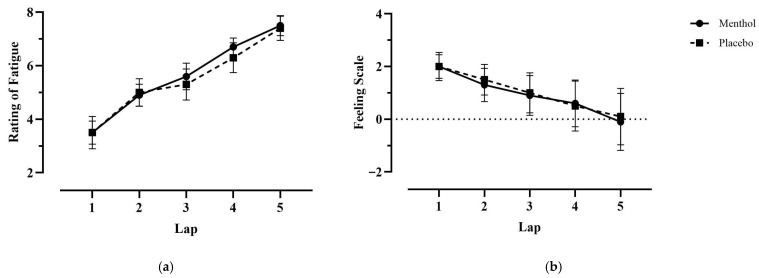
Relationship between rating of fatigue (ROF) (**a**), feeling scale (FS) (**b**), and laps (time) during the modified variable cycling test (M-VCT). Data are presented as means ± SEM (*n* = 10). ROF increased significantly from the beginning to the end of each trial. Mean ROF was not significantly different between MEN and PLA across laps (*p* = 0.560). FS gradually decreased from the beginning of the trial to the end; however, there were no differences between MEN and PLA (*p* = 0.670).

Rating of fatigue (ROF) significantly increased from the start of the trial to the end of lap 5 under both conditions (MEN, 3.50 ± 1.90 vs. 7.50 ± 1.18, *p* < 0.001; PLA, 3.50 ± 1.35 vs. 7.40 ± 1.43, *p* < 0.001) while feeling scale (FS) significantly decreased (MEN, 2.00 ± 1.70 vs. −0.10 ± 3.41, *p* = 0.002; PLA, 2.00 ± 1.41 vs. 0.10 ± 3.38, *p* = 0.008). Mean ROF and FS were not significantly different between trials, and a pairwise comparison per lap showed no significant difference in ROF or FS measurements at any time point between conditions (*p* > 0.05 across all laps) (Table 1, Figure 5).

### 4.7. Surface Electromyography (sEMG)

**Figure 6:**
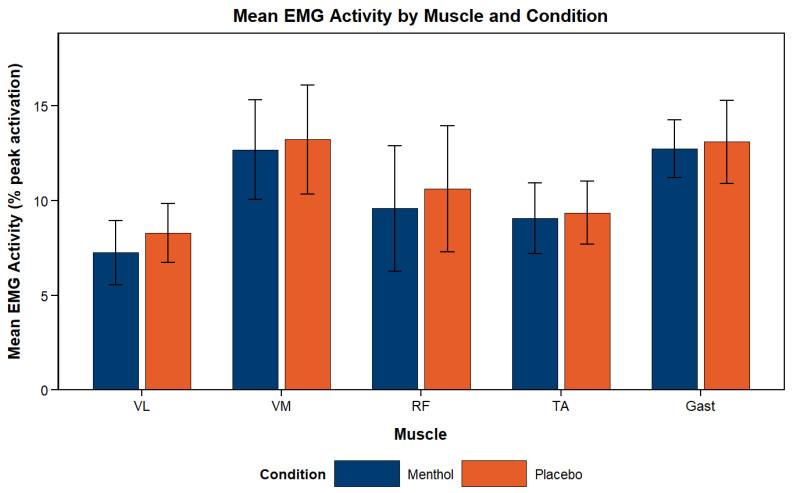
Mean surface electromyography amplitude (aEMG) during fixed resistance workload sections of the M-VCT per muscle. Data are presented as estimated marginal means ± SEM (*n* = 10). No significant main effect of condition was observed for any individual muscle (VL: *p* = 0.057; VM: *p* = 0.598; RF: *p* = 0.252; TA: *p* = 0.690; Gast: *p* = 0.562).

**Figure 7:**
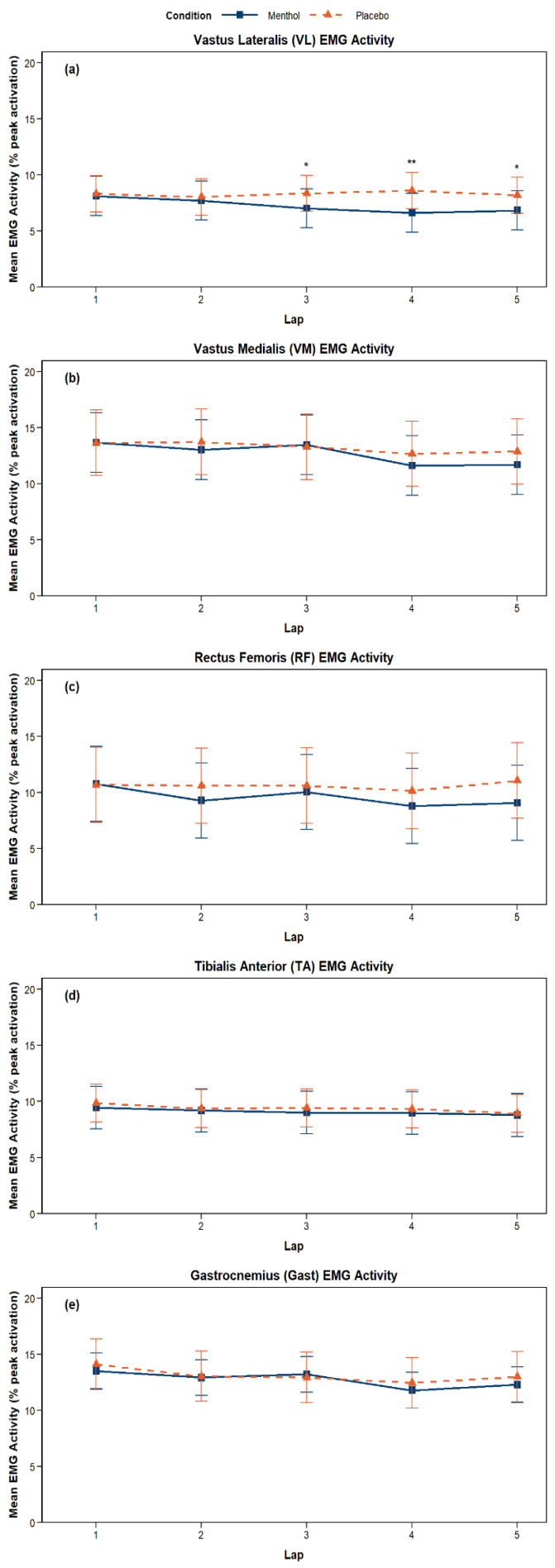
Estimated marginal mean (EMM) surface electromyography amplitude (aEMG) during fixed resistance workload sections of the M-VCT across the trial duration (lap). Each muscle (VL (**a**), VM (**b**), RF (**c**), TA (**d**), and Gast (**e**)) is represented with an individual plot. Laps 1–5 are represented on the x-axis, with MEN (blue squares, solid line) and PLA (orange triangles, dotted line) represented. Error bars for each time point are shown (SEM). Post hoc analyses were performed only on muscles with significant condition x lap interaction effects (VL & RF). MEN resulted in significantly reduced EMG activity than PLA during laps 3, 4, and 5 for the VL. No laps showed a significant difference between MEN and PLA for RF, other than a trend to significance for lap 5 (*p* = 0.053) * *p* < 0.05, ** *p* < 0.01.

A significant main effect of lap was observed for VM (*p* < 0.001), TA (*p* = 0.048), RF (*p* = 0.003), and Gast (*p* < 0.001), with a progressive decrease in EMG RMS amplitude for both MEN and PLA. Within each lap, measures were taken during five separate workloads. A significant main effect of workload, indicating increased EMG RMS amplitude as each set progressed, regardless of condition, was observed for VM (*p* = 0.012), TA (*p* < 0.001), and RF (*p* < 0.001). A trend toward a main effect of condition was observed only for VL (*p* = 0.057), with lower activation in MEN (7.25% peak activation ± 0.75 (SE)) versus PLA (8.29% peak activation ± 0.69 (SE)) (Figure 6). Significant condition × lap interactions were identified for VL (*p* = 0.007) and RF (*p* = 0.017), indicating lower activation in MEN across the testing time. Post hoc analyses showed significant differences between MEN and PLA on the final three laps for VL (lap 3: *p* = 0.037; lap 4: *p* = 0.003; lap 5: *p* = 0.035). For RF, no individual lap differed significantly, although a trend was observed on lap 5 (*p* = 0.053). No condition × workload interactions were observed for any muscle (Figure 7).

## 5. Discussion

This study demonstrates that menthol mouth rinsing enhances cycling performance in the heat with measurable changes in peripheral neuromuscular activation across trial laps, as assessed by surface electromyography (sEMG). Specifically, 10 trained adolescent cyclists produced higher power output, maintained higher cadence, and exhibited lower sEMG activation in the vastus lateralis (VL) and rectus femoris (RF) over time in the menthol (MEN) condition compared to the placebo (PLA) condition. While there appears to be an overall trend towards lower EMG activity in the menthol trials (*p* = 0.57 in VL), no muscles differed significantly when assessed independently across conditions. Collectively, these findings further explored the ergogenic effects of menthol MR in thermally stressful environments and suggest that some interaction with fatigue-induced alterations in neuromuscular activation patterns is generating the performance benefits. Menthol mouth rinse has formerly been linked to perceptual or centrally mediated mechanisms rather than peripheral [4,15,22], and thus the performance and sEMG changes observed in the current study may be reflective of the downstream effects of menthol MR.

Previous research has shown that menthol acts on TRPM8 receptors, inducing a cooling sensation without reductions in core temperature, improving thermal comfort and potentially delaying the onset of centrally mediated fatigue [15,16]. While our past work has demonstrated the performance-related benefit of using menthol mouth rinsing in the heat during a race-like cycling task [17], the present study extends these findings by replicating the experimental approach and incorporating sEMG measures to examine neuromuscular activation patterns, providing preliminary insight into the potential mechanisms that may underlie the performance changes measured in this population. Hence, the current findings align with these prior results, as menthol MR improved objective performance markers without changes in physiological strain [17,36]. Despite the increased mechanical work in MEN (higher power output), perceptual ratings of fatigue and affect did not differ between trials, supporting the hypothesis that menthol MR may dissociate perceptual strain from actual workload, enabling athletes to push harder without experiencing higher levels of exertion [4,22].

sEMG analysis revealed a significant main effect of time (lap) across trials, with a progressive decline in muscle activation consistent with accumulating neuromuscular fatigue (decrease in EMG amplitude over time). While no significant differences were observed in sEMG amplitude between the menthol and placebo conditions, visual trends suggest slightly lower muscle activation in the menthol condition across all muscles despite concurrent increases in cadence and power output. This pattern suggests that alterations in neuromuscular activation over time may reflect changes in fatigue-related responses, albeit of unknown origin, potentially involving a combination of central and/or peripheral contributions [13,15,22]. It is also noteworthy that a plausible explanation for the higher muscular activation during the PLA condition could indicate elevated compensatory drive (from the CNS) to maintain output in working muscles despite lower power output and performance [5,11] when compared to the MEN condition.

Furthermore, significant condition × lap interaction effects were detected in both the VL and RF. Post hoc analyses revealed that MEN resulted in significantly reduced EMG activity during laps 3, 4, and 5 for the VL compared to PLA, whereas no laps showed a significant difference between MEN and PLA for RF, except for a trend toward significance in lap 5 (*p* = 0.053). While many limb muscles play a vital role in the pedal stroke for power production and maintenance, significant effects were detected only in these two dominant muscles of the quadriceps. Since hamstring activation was not assessed, it remains unclear whether a more efficient pedalling strategy was maintained due to greater or altered engagement from the hamstrings, or if a shift occurred in the perceptual fatigue response profile, enabling reduced neural drive for the same or greater mechanical output, effectively creating a different trajectory of muscle recruitment over time with MEN. Although the sample size was relatively small, significant differences in EMG activity were detected and supported by moderate effect sizes, suggesting that the observed neuromuscular responses were meaningful within this cohort. The use of a highly trained population may have reduced inter-individual variability, increasing the sensitivity of the repeated-measures design. However, these findings should be interpreted as preliminary and hypothesis-generating, and future studies with larger sample sizes are required to confirm these results and determine their generalizability.

Nonetheless, these findings are in line with previous studies involving caffeine and carbohydrate mouth rinses, which have demonstrated enhanced endurance performance and increased fatigue tolerance with significant reductions in EMG activity during intense cycling tasks—an effect attributed to the activation of central neural circuits involved in motor control and reward through oral sensory stimulation [37]. The absence of elevated muscle recruitment in the menthol trial, despite enhanced mechanical output, strengthens the hypothesis that menthol does not exert its ergogenic effects via peripheral mechanisms. Instead, the preserved muscle activation profiles alongside stable thermoregulatory and metabolic responses (i.e., core temperature and heart rate) suggest that menthol MR acts centrally, likely by attenuating perceived thermal discomfort and sustaining motivation [17,22,24]. Future research directly measuring central mechanisms in each of these scenarios will further localize MR-induced neuromuscular changes. This interpretation aligns with central fatigue models under thermal strain, wherein performance decrements are primarily governed by perceptual and cognitive factors rather than peripheral muscle limitations [13]. Overall, menthol MR facilitated greater work output under heat stress with reduced apparent neuromuscular burden.

Given that the sample consisted of adolescent male cyclists, the EMG findings may also reflect age-specific neuromuscular characteristics. Youth athletes (trained) are typically more resistant to peripheral fatigue and more prone to central fatigue owing to greater reliance on oxidative metabolism and fatigue-resistant fibre profiles [3,38]. Moreover, it has previously been observed that children and adolescents are more susceptible to central fatigue of the knee extensors when compared to adults, as measured by voluntary activation [39,40]. Thus, the moderate reduction in sEMG amplitude with MR observed in the current study could represent a larger proportion of central fatigue changes when compared to an adult population [1,4,40]. This study also contributes to the emerging body of literature on adolescent athletic performance and thermoregulation. Despite their developmental stage, trained adolescent cyclists responded to menthol MR similarly to adult cohorts in terms of power output and cadence, suggesting that TRPM8-mediated cooling strategies are effective across age groups, provided that training status is equivalent.

### Limitations and Future Directions

Although this study provides novel insight, several limitations should be acknowledged. First, sEMG was limited to superficial muscles and could not resolve deeper neuromuscular dynamics or cortical excitability (CNS activity). Due to limited sensor availability, only muscles in the quadriceps and calves were assessed, leaving gaps in our understanding of the precise muscular recruitment patterns or strategy changes in pedalling technique that may occur in the hamstrings during the cyclical pedal stroke to impact neuromuscular efficiency over time. Second, the small sample size may have limited the statistical power to detect subtle changes in EMG patterns. Nonetheless, trained adolescent cyclists remain a novel and under-researched cohort, thereby fulfilling a clear research gap in performance investigations. Third, given the distinct sensory properties of menthol, the single-blind design may not have fully eliminated expectancy effects. The participants were informed only that they would receive different mouth-rinse conditions during a race-like cycling task and were asked to rank their preferences. Since blinding effectiveness was not formally assessed, flavour preference was only recorded for descriptive purposes and should not be interpreted as evidence that expectancy effects were controlled. Finally, while sEMG amplitude was normalized to individual peak values, muscle fibre type recruitment or changes in EMG frequency spectra were not assessed. Future work should incorporate frequency domain analyses and central measures (e.g., TMS, EEG) to further clarify menthol’s mechanism of action. Additionally, exploring sex differences and the effects of the menstrual phase in female athletes would enhance generalizability.

## 6. Conclusions

Menthol mouth rinsing significantly enhanced cycling performance in the heat and reduced sEMG activation over time in the quadriceps muscles (VL, RF) despite negligible changes in perceptual fatigue, cardiovascular, and thermoregulatory responses. It remains unclear if menthol acts via a perceptual and/or central nervous system-mediated mechanism. The findings of increased cadence and power output provide compelling evidence for the inclusion of menthol-based cooling strategies in thermoregulatory protocols for endurance athletes, especially when competing in hot conditions.

### Practical Applications

- **Athletes and coaches:** MEN MR represents a low-risk, non-thermal cooling strategy that may enhance cycling performance in hot conditions by supporting higher power output and cadence without increasing perceived fatigue. **Researchers:** These findings position MEN as a useful future model for examining sensory-driven modulation of neuromuscular fatigue under thermal stress, with relevance for age-specific responses

## Figures and Tables

**Table 1:** Mean values of physiological and performance variables during the menthol (MEN) and placebo (PLA) trials of the modified variable cycling test (M-VCT) (*n* = 10).

Variable	MEN	PLA	*p* Value	95% CI	ES
Total mean power (W)	177.1 ± 33.0	174.1 ± 32.1	0.002 *	[1.48 to 4.47]	1.42
1 s peak power (PPO) (W)	1150.8 ± 252.2	1125.7 ± 247.2	0.202	[−16.2 to 66.4]	0.44
6 s “acceleration” PPO (W)	441.0 ± 138.6	436.6 ± 96.7	0.933	[−110.62 to 119.42]	0.03
10 s “hard” PPO (W)	1030.1 ± 229.0	963.6 ± 188.3	0.138	[−25.99 to 158.99]	0.51
Final sprint PPO (W)	1116.9 ± 248.9	1110.1 ± 272.3	0.71	[−33.7 to 47.3]	0.12
Total mean cadence (RPM)	87.4 ± 5.1	84.5 ± 5.2	0.027 *	[0.42 to 5.37]	0.84
6 s “acceleration” mean cadence (RPM)	92.4 ± 12.4	90.6 ± 11.2	0.078	[−0.25 to 3.92]	0.63
10 s “hard” mean cadence (RPM)	107.4 ± 10.0	106.0 ± 9.4	0.022 *	[0.26 to 2.66]	0.87
Final sprint mean cadence (RPM)	123.5 ± 10.2	123.7 ± 10.5	0.857	[−3.06 to 2.59]	0.06
Trial distance (m)	21,727.5 ± 4004.5	21,404.1 ± 3884.2	0.005 *	[127.2 to 519.5]	1.18
Mean heart rate (bpm)	163.4 ± 15.2	162.5 ± 16.2	0.373	[−1.27 to 3.07]	0.3
Max heart rate (bpm)	185.3 ± 12.3	184.9 ± 11.8	0.653	[−1.54 to 2.34]	0.15
Mean core temperature (°C)	37.6 ± 0.3	37.7 ± 0.4	0.237	[−0.38 to 0.11]	0.4
Max core temperature (°C)	38.1 ± 0.3	38.1 ± 0.5	0.610	[−0.35 to 0.22]	0.17
Rating of fatigue (ROF)	5.64 ± 1.27	5.50 ± 1.43	0.56	N/A	N/A
Feeling scale (FS)	0.94 ± 2.43	1.02 ± 2.36	0.67	N/A	N/A

For performance, physiological and subjective data: data are presented as means ± SD. * *p* < 0.05. MEN, menthol; PLA, placebo; 95% CI, 95% confidence interval; ES, Cohen’s D effect size estimate; W, watt; PPO, peak power output; s, seconds; RPM, revolutions per minute; m, metres; bpm, beats per minute; °C, degrees Celsius.

**Table 2:** Mean values of muscle sEMG during the menthol (MEN) and placebo (PLA) trials of the modified variable cycling test (M-VCT) (*n* = 10).

Muscle sEMG (% Peak Activation)	MEN	PLA	*p* Value	95% CI	ES
Vastus lateralis (VL)	7.25 ± 0.75	8.29 ± 0.69	0.057	[−2.12 to 0.04]	0.48
Vastus medialis (VM)	12.7 ± 1.16	13.2 ± 1.27	0.598	[−2.78 to 1.7]	0.22
Rectus femoris (RF)	9.59 ± 1.47	10.62 ± 1.47	0.252	[−2.94 to 0.87]	0.42
Tibialis anterior (TA)	9.07 ± 0.81	9.36 ± 0.72	0.69	[−1.94 to 1.35]	0.16
Medial gastrocnemius (Gast)	12.7 ± 0.67	13.1 ± 0.97	0.562	[−1.73 to 1]	0.14

Data are presented as estimated marginal means ± SE. MEN, menthol; PLA, placebo; 95% CI, 95% confidence interval; ES, Cohen’s D effect size estimate.

## Data Availability

The original contributions presented in this study are included in the article. Further inquiries can be directed to the corresponding authors.

## Abbreviations

The following abbreviations are used in this manuscript:

MEN	menthol
PLA	placebo
MR	mouth rinse
TRPM8	transient receptor potential melastatin 8
M-VCT	modified variable cycle test
PO	power output (W)
PPO	peak power output (W)
RPM	revolutions per minute
HR	heart rate (bpm)
Tc	core body temperature (°C)
ROF	rating of fatigue
FS	feeling scale
sEMG	surface electromyography
VL	vastus lateralis
VM	vastus medialis
RF	rectus femoris
TA	tibialis anterior
Gast	gastrocnemius
